# Butyrate inhibits interleukin-17 and generates Tregs to ameliorate colorectal colitis in rats

**DOI:** 10.1186/s12876-016-0500-x

**Published:** 2016-07-30

**Authors:** Mingming Zhang, Qian Zhou, Robert G. Dorfman, Xiaoli Huang, Tingting Fan, Hao Zhang, Jun Zhang, Chenggong Yu

**Affiliations:** 1 Department of Gastroenterology, Nanjing Drum Tower Hospital, the Affiliated Hospital of Nanjing University Medical School, Nanjing University, Nanjing, China; 2 Department of Digestive Diseases, Huashan Hospital, Fudan University, Shanghai, China; 3 School of Life Sciences, Fudan University, Shanghai, China; 4 Institutes of Biomedical Sciences, Fudan University, Shanghai, China; 5 Northwestern University Feinberg School of Medicine, Chicago, IL USA; 6 Department of Gastroenterology, Nanjing Jiangbei People’s Hospital Affiliated to Southeast University Medical School, Nanjing, China; 7 State Key Laboratory of Pharmaceutical Biotechnology, School of Life Sciences, Nanjing University, Nanjing, China

**Keywords:** Butyrate, Inflammatory bowel disease, Cytokines, Th17, Treg

## Abstract

**Background:**

Butyrate is an energy source for colonocytes that is formed by bacterial fermentation of dietary fiber in the colon and that exerts broad anti-inflammatory activities. Although the administration of butyrate improves homeostasis in patients and ameliorates IBD (Inflammatory Bowel Disease)-related lesions and symptoms, the anti-inflammatory mechanisms of butyrate still remain unclear. To explore the impact of butyrate on Treg (Regulatory T cell)/Th17 (T helper 17 cell) differentiation and colitis in rats.

**Methods:**

The effect of butyrate on the expression of markers related to both Tregs and Th17 cells were determined in human monocytes as well as a rat model of colitis induced by 2,4,6-trinitrobenzene sulfonic acid. Rats were treated with butyrate in vivo, whereas the rat splenocytes and human monocytes were treated in vitro.

**Results:**

We found that butyrate administration increased peripheral blood Treg cell levels as well as plasma levels of anti-Th17 cytokines (IL-10 and IL-12). Butyrate administration further suppressed IL-17 levels in both plasma and colonic mucosa, and ameliorated colonic colitis lesions in rats. This promotion of Treg activity and inhibition of IL-17 release was also observed in human venous monocytes and rat splenocytes in vitro.

**Conclusions:**

Our results suggest that butyrate plays a key role in regulating the Treg/Th17 balance and ultimately protects the colon mucosa against the development of IBD.

## Background

Inflammatory bowel disease (IBD) consists of a group of disorders characterized by recurrent inflammation in the gastrointestinal tract. The two most common forms of IBD are ulcerative colitis (UC) and Crohn’s disease (CD). While the etiology of IBD remains uncertain, it has been hypothesized that an undesired intestinal mucosal immune response to luminal contents contributes to the onset of IBD in a genetically predisposed patient [1].

CD4^+^ T helper (Th) cells regulate immunity and inflammation through antigen-dependent activation and cytokine-dependent differentiation into functional T cell subsets. T helper 17 (Th17) cells are unique pro-inflammatory Th cells identified by retinoic acid receptor-related orphan receptor gamma t (RORγt) and interleukin-17 (IL-17) [2]. The IL-23/Th17/IL-17 pathway plays an important role in regulating IBD, and studies have found that Th17 levels are increased in both the colonic mucosa and serum of IBD patients [3]. Other T cells that differentiate from Th cells include regulatory T cells (CD4^+^CD25^+^FoxP3^+^ Treg), which play a key role in modulating the immune response [4]. Tregs are defined by the expression of both surface CD4^+^CD25^+^ and the intracellular transcription factor, FoxP3, which plays a key role in regulating Treg activity [5]. Tregs regulate the homeostasis of the intestinal immune system by promoting anti-inflammatory cytokine production, including interleukin-10 (IL-10), and exerting dominant negative regulation of other T helper cells such as Th17 [6]. Tregs both produce and respond to TGF-β, an anti-inflammatory cytokine that plays an important role in maintaining Treg activity [7]. IL-6, an important signaling protein for maintaining the Treg/Th17 balance, suppresses Treg maturation and promotes a predominantly Th17 mediated pro-inflammatory response [8].

Short-chain fatty acids (SCFAs) are formed by bacterial fermentation of non-starch polysaccharides (NSP), such as dietary fiber in the colon [9]. Butyrate is mostly produced by *Faecalibacterium prausnitzii* (*F. prausnitzii*) and serves as an energy source for colonocytes. It appears to exert a promising anti-inflammatory effect by influencing immune cell migration, cytokine expression, and other cellular processes (eg. proliferation, activation and apoptosis) [10]. Studies in UC patients have suggested that both the administration of butyrate and the stimulation of luminal butyrate production by the ingestion of dietary fiber have an ameliorating effect on intestinal inflammation and related symptomology [11].

Although the administration of butyrate improves IBD-related lesions and symptoms, the exact anti-inflammatory mechanism remains unclear, and its impact on Treg/Th17 differentiation has not yet been examined. Therefore, the hypothesis of this study is that the administration of butyrate can ameliorate lesions and symptoms of colorectal UC in rats by inhibiting Th17 generation and promoting Tregs.

## Methods

### Patients

Both patients and healthy participants were recruited by the Affiliated Hospital of Nanjing University and exposed to the same diet and living environment. None of the participants used probiotics, antibiotics, or sulfasalazine in the preceding two months. UC diagnosis was based on clinical, endoscopic, and histological criteria, and UC disease activity was defined using the Simple Clinical Colitis Activity Index (SCCAI) on the date of fecal sampling [12]. The characteristics of all participants are shown in Table 1.

**Table 1:** Characteristics of the study population

Characteristics	Control (*N* = 10)	UC (*N* = 7)	*p* Value
Male/female (%)	5/5(50/50)	3/4(43/57)	p > 0.05
Median (IQR) age (years)	47(31–50.5)	47(31–50.5)	p > 0.05
Median (IQR) duration of disease (years)	NA	5(2–6.5)	NA
Median (IQR) BMI	25(23–26.5)	26(21–27)	p > 0.05
Medication	NA		NA
5-Aminosalicylic acid (%)		7(100)	
Azathioprine (%)		4(57)	
Corticosteroids (%)		2(29)	
Anti-TNF (%)		1(19)	
Disease activity	NA		NA
Remission (SCCAI Score < 5)		3(43)	
Active (SCCAI Score ≥ 5)		4(57)	

Groups were compared by non-parametric analyses

*BMI* body mass index, *NA* not applicable

*p* < 0.05 was considered significant

### Modeling of colorectal colitis in rats and treatment

The rats (Sprague–Dawley, 7 weeks, males) were induced by TNBS administration [13]. Briefly, the TNBS (Sigma) solution was slowly administered in the colon (100 mg/kg body weight) via a 4.7 mm-diameter catheter. The control was administered with vehicle. After TNBS-administration, the rats were gavaged daily with sodium butyrate (Sigma) solution (0.5 mM/kg body weight) for 20 consecutive days. The control was administered with vehicle. The rats were weighed at the indicated time and killed using ether exposure at day 21. The colon specimens were stained with hematoxylin and eosin, and lesions were analyzed using the modified Neurath Scoring criteria [14] (briefly, 0 = no inflammation; 1 = very low level of leucocyte infiltration; 2 = low level of leucocyte infiltration; 3 = high level of leucocyte infiltration, high vascular density, thickening of the colon wall; 4 = transmural leucocyte infiltrations, loss of goblet cells, high vascular density, thickening of the colon wall).

### Short-chain fatty acids (SCFAs) assay

Fresh faecal samples were collected and stored in at −80 °C. Faecal samples were mixed with water and centrifuged. The supernatant was filtered and mixed with ether and sulfuric acid. After high speed centrifugation, the ether layer was collected and measured in the Agilent 6890 N Gas Chromatograph Machine for SCFA concentrations.

### Immunohistochemistry

Colon specimens were fixed in 4 % formalin and embedded in paraffin. The sections were were incubated with rabbit anti-rat IL17 antibodies (Abcam) and then treated with immunoperoxidase using the DAB kit (Zsbio). Sections were scored in a blind manner using a protocol modified from Brown & Wahl [15].

### Peripheral blood mononuclear cell (PBMC) culture

PBMCs were isolated from the venous blood of healthy donors [16]. The cells were subsequently suspended in complete medium (2 × 10^6^ cells/ml) and seeded in a 24-well plate (2 × 10^6^ cells/well). PBMCs were then treated with PBS and different concentrations of sodium butyrate (Sigma), respectively, for 72 h in 24-well plates pre-coated with UV-irradiated *E. coli* at a PBMC:bacteria ratio of 1:10. After 72 h, the culture supernatant from the PBMCs was collected and stored at −80 °C for cytokine analysis. PBMCs were used for flow cytometry.

### FCM (flow cytometry) analysis of Treg cells

Mononuclear cells were isolated from blood using Ficoll-Isopaue density gradient centrifugation (Ficoll-Paque, MP Biomedicals). FCM followed routine procedures, and cells were labeled with FITC anti-CD4 (eBioscience), APC anti-CD25 (eBioscience) and PE anti-Foxp3 (eBioscience).

### Primary splenocyte culture

Untreated 7-week-old SD rats were sacrificed using cervical dislocation following ether exposure. The splenocytes were incubated with recombinant human TGF-β (2 ng/ml, Peprotech) and recombinant rat IL-6 (20 ng/ml, Peprotech) at 37 °C for 72 h, whereas the control was treated with vehicle. Experimental group cells were treated with PBS or different concentrations of sodium butyrate (Sigma) in addition to TGF-β and IL-6 [17].

### Primary splenocyte and bone marrow-derived dendritic cell (BMDC) culture

Untreated 7-week-old SD rats were sacrificed using cervical dislocation following ether exposure. The splenocytes were incubated with recombinant human TGF-β (2 ng/ml, Peprotech) and recombinant rat IL-6 (20 ng/ml, Peprotech) at 37 °C for 72 h [17]. Immature BMDCs were isolated using a protocol modified from Inaba et al. [18], in the presence of recombinant rat IL-4 (10 ng/ml, Peprotech) and recombinant rat granulocyte-macrophage colony-stimulating factor (GM-CSF, 10 ng/ml, Peprotech).

### ELISA assay and western blotting

Cytokines (IL-10, IL-17A, IL-12 p70, TGF-β1, IL-6 and IL-23) were measured using a commercially available ELISA kit (Bender: IL-10, IL-17A, IL-12 p70 kits; SABC: IL-23 kit; eBioscience: TGF-β1, IL-6 kits) according to the manufacturer’s instructions. For western blotting, cells were lysed using 0.5 % NP40 lysis buffer and proteins were blotted following standard protocol. Antibodies to RORγt (Abcam) and actin (GenScript Corp) were purchased commercially.

### Statistics

Data was expressed as the mean ± standard error of the mean (SE). The data was analyzed with one-way ANOVA followed by a post hoc Duncan test (SPSS 17.0). *P* < 0.05 was considered significant.

## Results

### Intestinal fatty acid levels

**Fig. 1:**
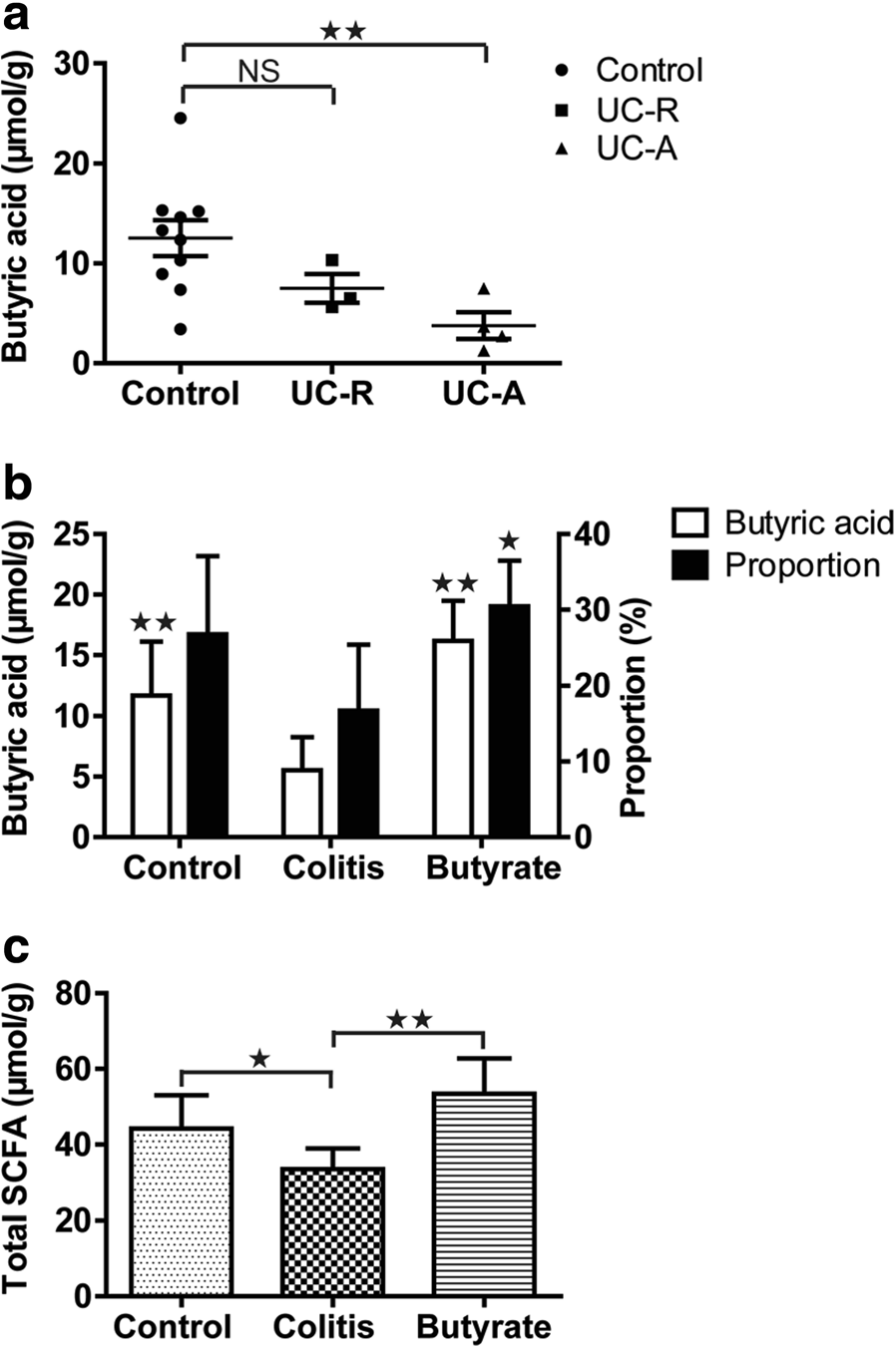
Intestinal fatty acid levels. Human fecal butyrate concentration (**a**). Rat fecal butyric acid concentration and percentage of total SCFA content (**b**). Rat fecal total SCFA content (**c**). Data are the mean ± SE. *n* = 5–7. *P < 0.05; **P < 0.01; NS: No Significance. UC-R, remission phase of ulcerative colitis; active phase of ulcerative colitis

Ulcerative colitis patients had a net concentration of butyric acid that was significantly lower than that of healthy controls (Fig. 1a). Moreover, rats in the TNBS-treated colitis group had significantly lower net concentrations of butyric acid and total SCFA concentrations than did rats in the control group (Fig. 1b-c). Following administration of sodium butyrate, fecal concentration of butyric acid, total SCFA, and the percentage of butyric acid were higher in the butyrate group than in the colitis group (Fig. 1b-c).

### Body weight, colon histology and blood cytokine production

**Fig. 2:**
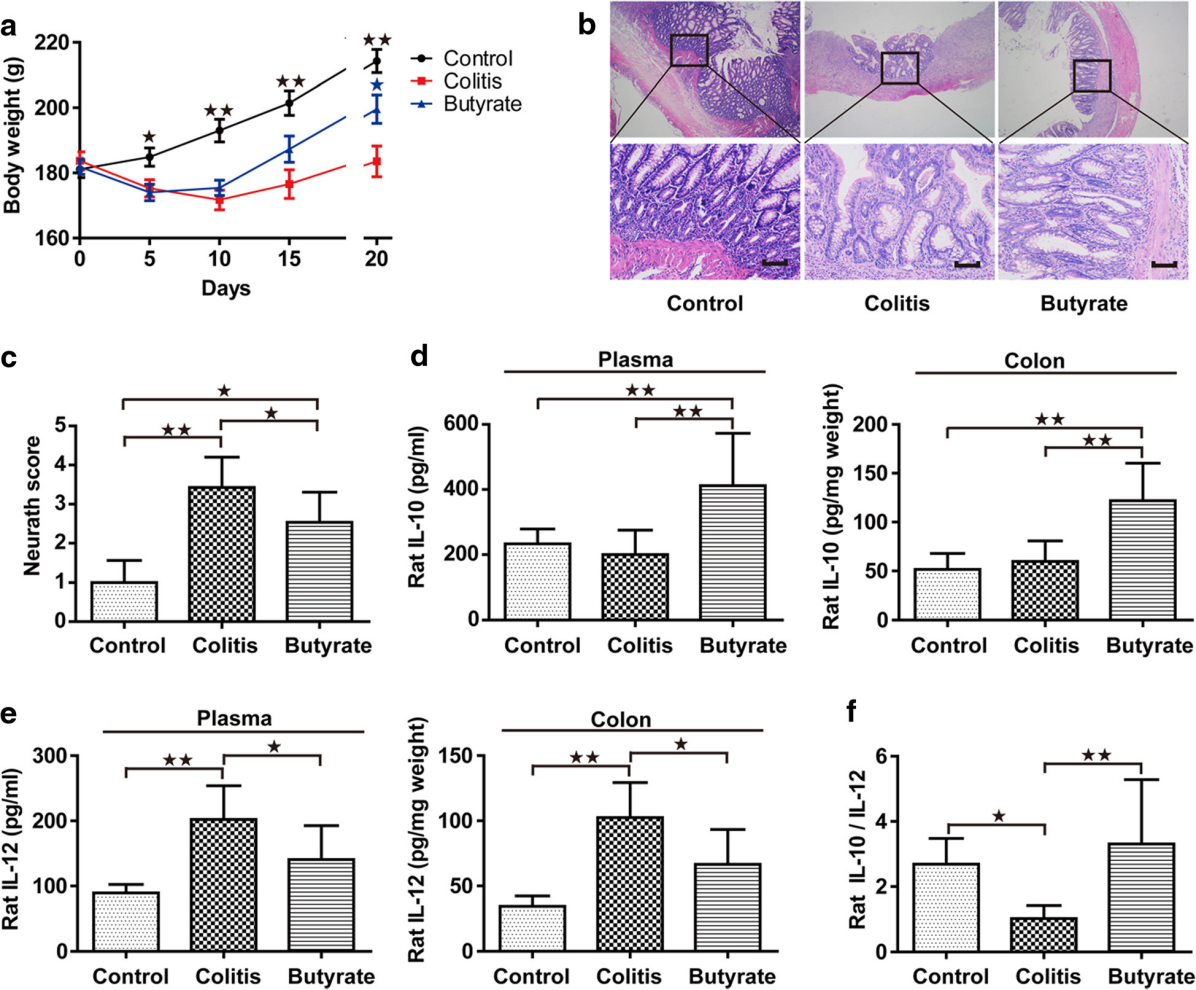
Body weight, colon histology and cytokines in rats. Body weight (**a**). Colon histology, upper and lower panel magnifications are × 40 and × 200, respectively. Scale bars, 200 μm (**b**). Colon Neurath score (**c**). IL-10 of plasma and colon (**d**). IL-12 of plasma and colon (**e**). Plasma IL-10/IL-12 (**f**). Data are the mean ± SE. *n* = 5–7. *P < 0.05; **P < 0.01

Rats in the TNBS-treated colitis group had significantly smaller weight gain, as well as more severe inflammation and higher colon Neurath scores than did rats in the control group (Fig. 2a-c). Butyrate administration significantly ameliorated the weight loss, increased colon inflammation, and higher Neurath scores observed in rats within the colitis group (Fig. 2a-c). Butyrate-treated rats only displayed mild mucosal and/or submucosal inflammation with a relatively low level of neutrophil infiltration and mild edema (Fig. 2b). Plasma cytokines, such as IL-12, IL-10 and the IL-10/IL-12 ratio, can be used to assess systemic levels of inflammation [19]. Plasma levels of IL-10 and the IL-10/IL-12 ratio were higher in the butyrate group than in the colitis group (Fig. 2d and f), but IL-12 levels were lower. The colonic cytokine results were consistent with the plasma results.

### Treg analysis in rats

**Fig. 3:**
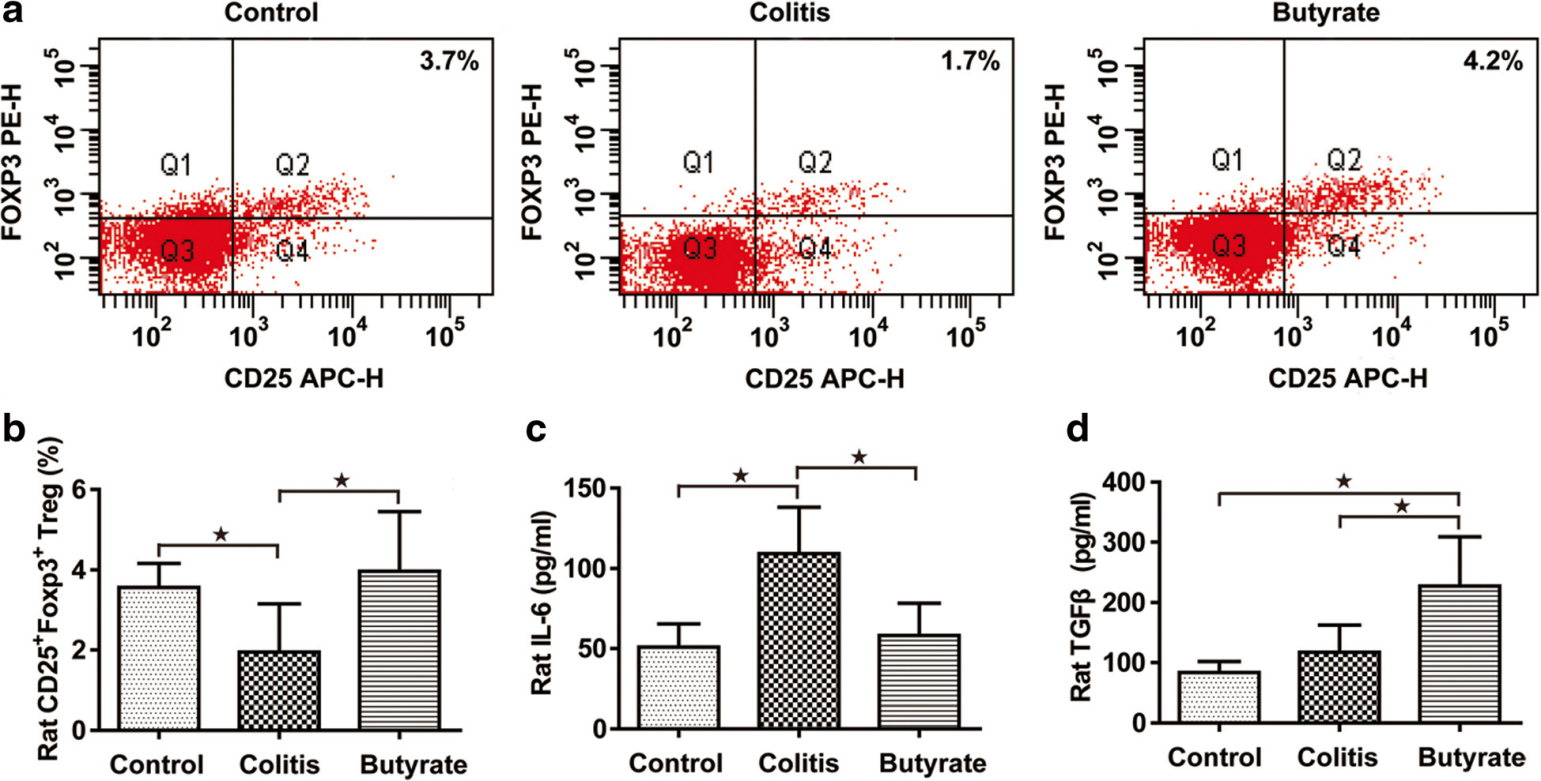
Treg analysis in rats. The percentage of CD4^+^CD25^+^Foxp3^+^ Tregs (**a**). Quantified CD4^+^CD25^+^Foxp3^+^ Tregs (**b**). Plasma IL-6 (**c**). Plasma TGF-β (**d**). Data are the mean ± SE. *n* = 5–7. *P < 0.05; **P < 0.01

In vivo studies demonstrated a significantly lower percentage of CD25 + Foxp3+ Tregs in the peripheral blood of rats from the TNBS-treated colitis group than in the control group (Fig. 3a and b). Following treatment with butyrate, the percentage of CD25 + Foxp3+ Tregs in the peripheral blood increased (Fig. 3a and b). With sufficient IL-6, TGF-β can stimulate native T cells to differentiate into Th17 cells [2]. However, without sufficient IL-6, TGF-β stimulates native T cell differentiation into Tregs [20]. As CD25 + Foxp3+ Treg frequencies increased following butyrate treatment, we examined the level of IL-6 and TGF-β in rat plasma. Plasma levels of IL-6 in the colitis group were significantly higher than those in the control group, which was consistent with the results of the CD25 + Foxp3+ Treg analysis in rat peripheral blood cells (Fig. 3c). Nonetheless, there was no significant difference in TGF-β levels between the two groups (Fig. 3d). Butyrate treatment, however, resulted in significantly lower plasma levels of IL-6 (Fig. 3c) and significantly higher levels of TGF-β (Fig. 3d) when compared to the colitis group.

### Th17 analysis of rats

**Fig. 4:**
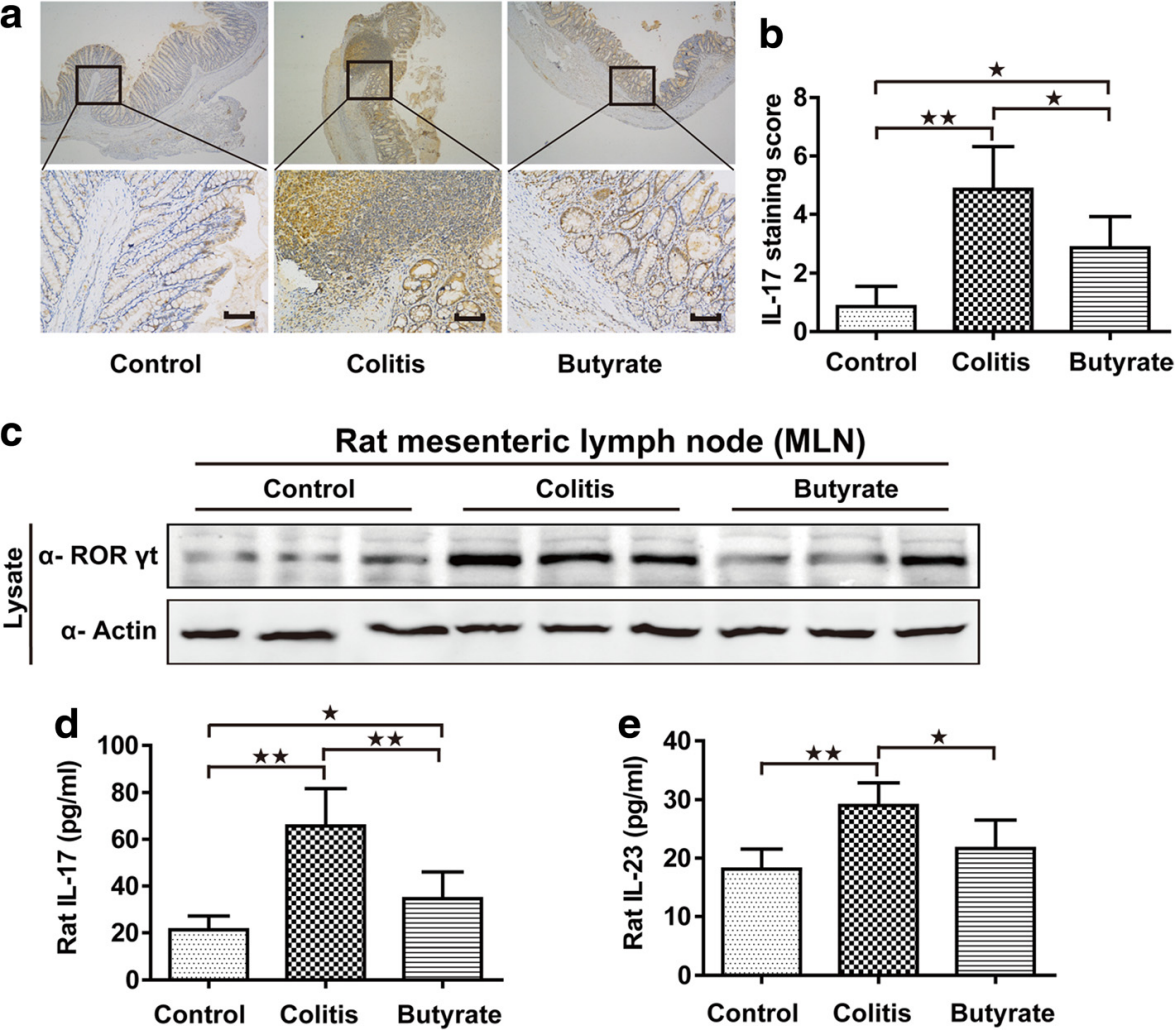
Th17 analysis in rats. IL-17 immunohistochemical staining in the colon; upper and lower panel magnifications are × 40 and × 200, respectively. Scale bars, 200 μm (**a**). Quantified IL-17 immunohistochemical staining in colon (**b**). Immunoblotting for RORγt in mesenteric lymph nodes, shown are representative western blot results of three rats (**c**). Plasma IL-17 (**d**). Plasma IL-23 (**e**). *n* = 5–7. Data are the mean ± SE. *n* = 7. *P < 0.05; **P < 0.01

IL-6 and TGF-β can stimulate native T cell differentiation into Th17 cells, which in turn produce and secrete IL-17 [2]. Th17 cells are identified by RORγt and IL-17, and IL-17 is detectable in both plasma and colon tissue in rats. IL-17 levels were found to be greater in the colitis group than in the control group, and decreased following butyrate treatment (Fig. 4a-b and d). Accordingly, butyrate treatment also decreased the colitis related RORγt increase in mesenteric lymph node (MLN) (Fig. 4c). IL-23 acts as an upstream regulator of Th17 cells and is critical for maintaining the stability and activation of Th17 cells [3]. Plasma IL-23 levels were increased in the colitis group, and likewise decreased following butyrate treatment (Fig. 4e).

### Treg and Th17 cell differentiation in vitro

**Fig. 5:**
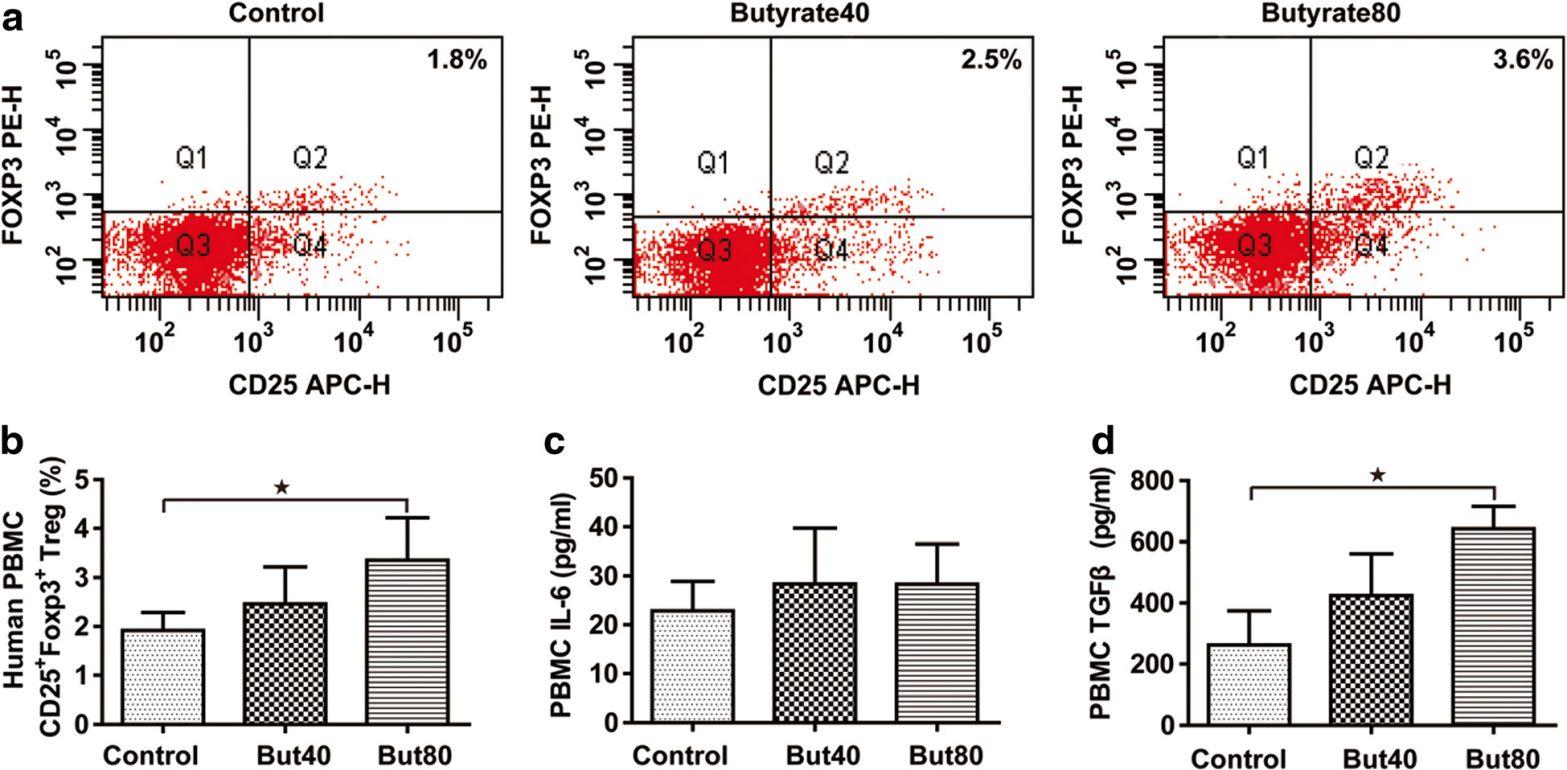
Treg cell differentiation in vitro. The percentage of CD4^+^CD25^+^Foxp3^+^ Tregs (**a**). Quantified CD4^+^CD25^+^Foxp3^+^ Tregs (**b**). Culture supernatant IL-6 (**c**). Culture supernatant TGF-β (**d**). Data are the mean ± SE. *n* = 4. *P < 0.05; **P < 0.01. But40, 40 μM sodium butyrate. But80, 80 μM sodium butyrate

**Fig. 6:**
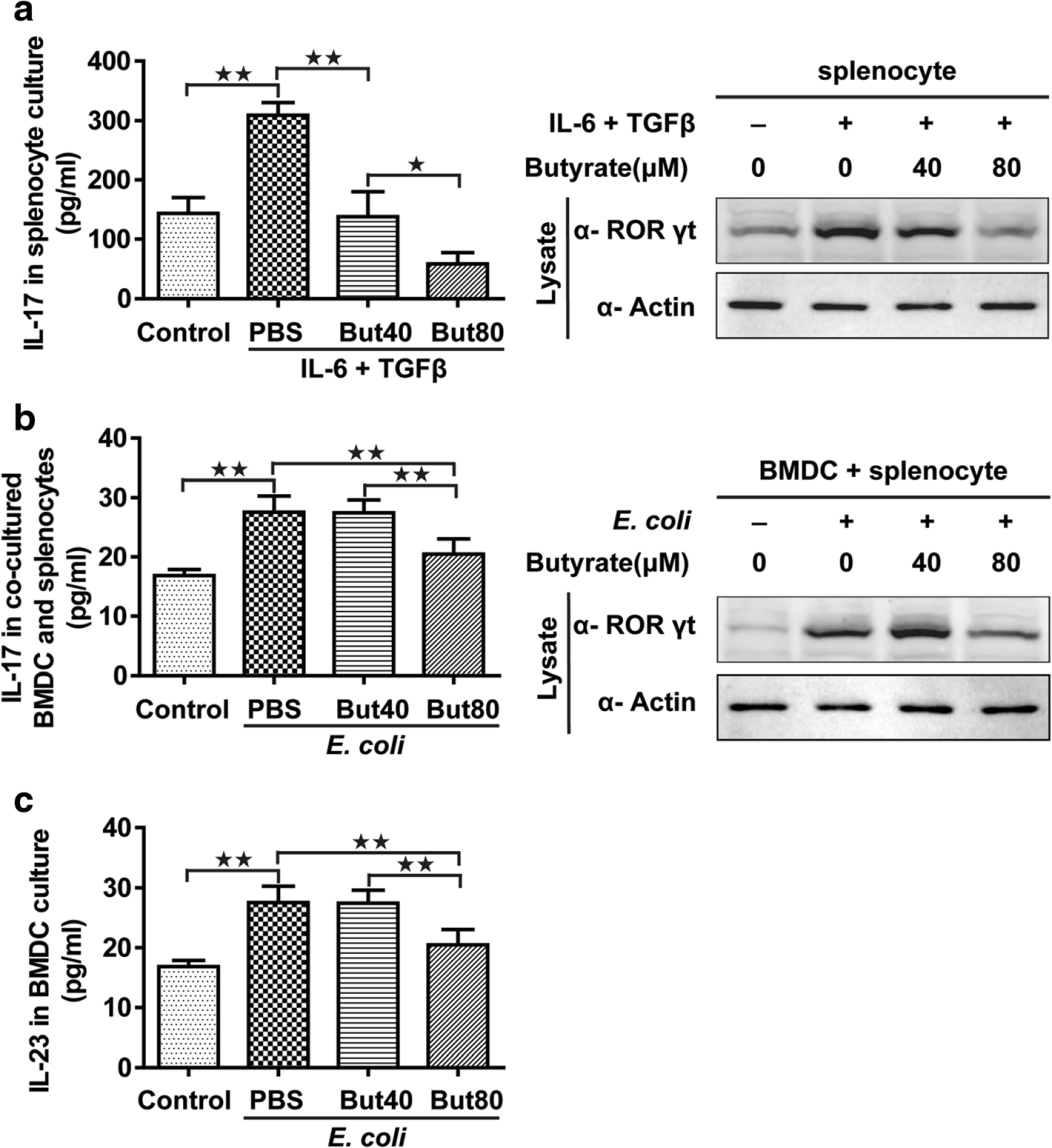
Th17 cell differentiation in vitro. IL-17 release from rat splenocytes (left) and immunoblotting for RORγt in rat splenocytes (right) in vitro (**a**). IL-17 release from co-cultured BMDCs and splenocytes (left) and immunoblotting for RORγt in co-cultured BMDCs and splenocytes (right) in vitro (**b**). IL-23 released from the BMDCs with UV-irradiated *E. coli* stimulation (**c**). Data are the mean ± SE. *n* = 4. *P < 0.05; **P < 0.01. But40, 40 μM sodium butyrate. But80, 80 μM sodium butyrate

We performed in vitro Treg Th17 analysis using human PBMCs in order to verify the in vivo experiments. The in vitro studies demonstrated that the CD4^+^CD25^+^Foxp3^+^ subpopulation in the PBMCs was consistently up-regulated by butyrate treatment in a dose-dependent manner (Fig. 5a-b). IL-6 and TGF-β can stimulate native T cell differentiation into Th17 cells [2] and immature bone marrow dendritic cells (BMDCs) can be activated by lipopolysaccharide (LPS) from *E.coli* to secrete IL-23, which plays an important role in maintaining the stability and function of Th17 cells [21]. In vitro, IL-6 and TGF-β treatment significantly increased IL-17 and RORγt levels from rat splenocytes, especially in comparison to the non-stimulated control group (Fig. 6a). Moreover, IL-17 and RORγt levels were significantly lower in cells cultured with butyrate in a dose-dependent manner than in the PBS group (Fig. 6a). In vitro, UV-irradiated *E.coli* caused a significant increase in IL-23 secretion in the immature BMDCs in comparison to the non-stimulated control group (Fig. 6c). Additionally, IL-23 secretion was significantly lower in cells cultured with 80 μM butyrate than in the PBS group (Fig. 6c). UV-irradiated E.coli in the PBS group further caused a significantly increase in IL-17 and RORγt levels when splenocytes and BMDCs were co-cultured (Fig. 6b). UV-irradiated *E. coli* -treated cells incubated with 80 μM butyrate expressed significantly less IL-17 and RORγt levels (Fig. 6b).

## Discussion

The major finding in this study was that butyrate exerted potent effects that ameliorated colitis lesions in a rat model by influencing Th cell differentiation and activation. This study proposes several possible mechanisms for these effects, including: 1) the inhibition of inflammatory Th17 cell activation and cytokine (IL-17) release, 2) the promotion of Treg cell differentiation and 3) alterations in the ratio in inflammatory cytokines (i.e., IL-10/IL-12) released by monocytes.

*Firmicutes* is a well-known butyrate-producing bacterium and several studies have confirmed that a reduction in *F. prausnitzii* and its product in IBD patients is inversely correlated with disease scores [22, 23]. Butyrate-producing microbiotic pharmabiotics have been shown to be effective in TNBS models and are destined for clinical trials [24]. Consistent with previous research in colitis models, our study confirmed that UC patients had lower fecal butyrate concentrations than did the control group. Our study utilized butyrate concentrations recommended by Di Sabatino et al. [25] and found that oral administration of butyrate resulted in increased percentage of butyric acid, fecal concentration of butyric acid, and total SCFA. This resulted in improved health status, including increased weight gain, lower colonic inflammation, and lower Neurath scores in the butyrate group rats as compared to the colitis group. These results were further confirmed by cytokine testing.

Th17 cells are a unique proinflammatory Th cell subset identified by RORγt and IL-17. Since the discovery of Th17, a number of studies have suggested that the IL-23/Th17/IL-17 pathway plays an important role in nearly all major autoimmune syndromes including IBD [3]. Studies have found that IL-17 levels are increased in both the colonic mucosa and serum of IBD patients, and IL-17 blocking therapy is being tested in patients with IBD [26, 27]. Serum concentration of total SCFAs in rodents is around 0.1 ~ 1 mM, among which only 10 % are butyrate [28, 29]. Therefore, the serum concentration of butyrate is estimated to be approximately 100 μM at most. Under physiological concentration of butyrate, we found that the high levels of RORγt and IL-17 caused by TNBS were both significantly reduced by butyrate treatment, suggesting their protective nature in the onset of colitis. IL-6 signaling is required for Th17 cell lineage commitment and its differentiation is enforced by TGF-β [2]. IL-23 is an essential upstream regulator of Th17 cells that maintains Th17 activity and function, and immature bone marrow dendritic cells (BMDCs) can be activated by lipopolysaccharide (LPS) from *E.coli* to secrete IL-23 [3, 21]. Our study found that plasma levels of IL-23 and IL-6 were significantly increased by TNBS treatment and reduced by butyrate administration. This suggests that the IL-23/Th17/IL-17 pathway is an effective target for butyrate treatment in the setting of inflammatory colitis. Our in vitro experiments in rat splenocytes and BMDCs further confirmed this in vivo data. When splenocytes differentiated into Th17 cells in vitro, we demonstrated a similar cytokine release profile to that observed in colitis rats. Only a high dose of butyrate (80 μM) could suppress the secretion of IL-23 by BMDCs and the secretion of IL-17 by the splenocytes when co-cultured with BMDCs. Consistent with IL-17 levels from cultured splenocytes, RORγt levels in splenocytes also decreased following butyrate treatment. Taken together, this suggests that butyrate inhibits Th17 differentiation.

Regulatory T cells (Treg) also differentiate from Th cells in presence of TGF-β [4]. Tregs maintain homeostasis by producing anti-inflammatory cytokines such as IL-10 and exert important negative regulation of Th17 cells. Impaired function of IL-10 and the IL-10 receptor are associated with aggressive IBD [6]. The insufficiency of Tregs in germ-free mice can be restored via a high-fiber diet induced by butyrate in vivo. This is corroborated by in vitro studies demonstrating that microbes producing butyrate stimulate the differentiation of Tregs and exert anti-inflammatory activity in the intestinal mucosa of a mouse model [30, 31]. Consistent with previous results, our in vivo study demonstrated butyrate treatment increased both the percentage of Treg and the levels of IL-10, suggesting that the protective effect of butyrate on intestinal inflammation was correlated with the level of IL-10. TGF-β, together with IL-6, can stimulate native T cells to differentiate into Th17 cells [2]. Yet without sufficient IL-6, TGF-β stimulates native T cells differentiation into Tregs [20]. Increased levels of Tregs in rat peripheral blood cells were found in combination with increased plasma levels of TGF-β, whereas butyrate treatment decreased IL-6 levels. The in vitro experiments in human PBMCs further confirmed this in vivo data. A high dosage of butyrate (80 μM) could promote secretion of TGF-β by PBMCs and increased Treg frequencies in PBMCs.

## Conclusions

In summary, a fine balance exists between Treg and Th17 cells in a healthy state. The same Th cell pool that generates Treg is also capable of producing Th17 cells, and this is coordinated by cytokines including IL-10, TGF-β and IL-6. Our results suggest that butyrate, a well-known metabolite, played a key role in regulating this Treg/Th17 balance and in turn yielded important insights for the treatment of IBD.

## Abbreviations

BMDC, bone marrow DC cells; CD, crohn disease; IBD, inflammatory bowel disease; IL, interleukin-23; LPS, lipopolysaccharide; PBMC, peripheral blood mononuclear cell; RORγt, retinoic orphan receptor γt; SCFA, short chain fatty acid; TGF-β, transforming growth factor β; Th17, helper T lymphocyte 17; TNBS, 2,4,6-trinitrobenzenesulfonic acid; UC, ulcerative colitis
